# Hyperhomocysteinemia Alters Retinal Endothelial Cells Barrier Function and Angiogenic Potential via Activation of Oxidative Stress

**DOI:** 10.1038/s41598-017-09731-y

**Published:** 2017-09-20

**Authors:** Riyaz Mohamed, Isha Sharma, Ahmed S. Ibrahim, Heba Saleh, Nehal M. Elsherbiny, Sadanand Fulzele, Khaled Elmasry, Sylvia B. Smith, Mohamed Al-Shabrawey, Amany Tawfik

**Affiliations:** 1 Department of Oral Biology and Anatomy, Dental College of Georgia, Augusta University, Augusta, GA USA; 2 James and Jean Culver Vision Discovery Institute, Medical College of Georgia (MCG), Augusta University, Augusta, USA; 3 Department of Biochemistry, Faculty of Pharmacy, Mansoura University, Mansoura, Egypt; 4 Department of Cellular Biology and Anatomy, MCG, Augusta University, Augusta, GA USA; 5 Department of Ophthalmology, MCG, Augusta University, Augusta, GA USA; 6 Department: Orthopedic Surgery, MCG, Augusta University, Augusta, GA USA

## Abstract

Hyperhomocysteinemia (HHcy) is associated with several human visual disorders, such as diabetic retinopathy (DR) and age-related macular degeneration (AMD). Breakdown of the blood-retinal barrier (BRB) is linked to vision loss in DR and AMD. Our previous work revealed that HHcy altered BRB in retinal endothelial cells *in vivo*. Here we hypothesize that homocysteine (Hcy) alters retinal endothelial cell barrier function and angiogenic potential via activation of oxidative stress. Human retinal endothelial cells (HRECs) treated with and without different concentrations of Hcy showed a reduction of tight junction protein expression, increased FITC dextran leakage, decreased transcellular electrical resistance and increased angiogenic potential. In addition, HRECs treated with Hcy showed increased production of reactive oxygen species (ROS). The anti-oxidant N-acetyl-cysteine (NAC) reduced ROS formation and decreased FITC-dextran leakage in Hcy treated HRECs. A mouse model of HHcy, in which cystathionine-β-synthase is deficient (*cbs* ^−/−^), was evaluated for oxidative stress by dichlolorofluorescein (DCF), dihydroethidium (DHE) staining. There was a marked increase in ROS production and augmented GSH reductase and antioxidant regulator NRF2 activity, but decreased antioxidant gene expression in retinas of hyperhomocysteinemic mice. Our results suggest activation of oxidative stress as a possible mechanism of HHcy induced retinal endothelial cell dysfunction.

## Introduction

The blood retinal barrier (BRB) is a highly selective barrier that regulates the transport of substances between the circulating blood and the neural retina and maintains neural retina health by providing essential nutrients and eliminating toxins and metabolic products^1^. Thus, disruption of BRB integrity is implicated in the pathology of many ocular diseases such as diabetic retinopathy (DR) and age-related macular degeneration (AMD). The BRB consists of inner and outer components; the inner BRB (iBRB) is formed by complex tight junctions between retinal capillary endothelial cells while the tight junctions of retinal pigment epithelial (RPE) cells form the outer BRB (oBRB)^2^. This study is focused on the inner blood retinal barrier. Retinal endothelial cells (RECs) contribute to the blood-retinal barrier that protects the retina from circulating molecular toxins, pro-inflammatory leukocytes and microorganisms. In addition, the RECs supply oxygen and nourishment to the metabolically active retina and allow circulating cells to maintain the vasculature and protect the retina from potential pathogens. If RECs are unable to fulfill these functions the retina is vulnerable to retinal vascular leakage, neovascularization and trafficking of inflammatory cells and microorganisms^3^. Damage to endothelial cells, followed by retinal vascular leakage and macular edema, are considered the earliest and most significant changes in major eye diseases such as DR, retinopathy of prematurity, retinal vein occlusion and uveitis^4^.

A large body of evidence emphasizes the significant role of oxidative stress in retinal vascular hyperpermeability and barrier dysfunction. Reactive oxygen species (ROS) have been shown to modify the molecular structure and morphology of endothelial tight junctions^5^. Homocysteine (Hcy) is a sulfur containing amino acid that is reported to decrease vascular activity by inducing oxidative stress^6,7^. Furthermore, Hcy is reported to alter the vascular phenotype and impair angiogenesis via oxidative stress^8,9^. Hcy is produced as an intermediate during metabolism of the essential amino acid methionine. Based on the methionine level, Hcy is either catabolized to cysteine by trans-sulphuration, a process catalyzed by cystathionine β-synthase (cbs), or recycled to produce methionine by transmethylation^8^. The methionine cycle is complex, involving the production of several intermediates implicated in redox homeostasis. These include glutathione and hydrogen sulfide (H_2_S), which regulate cellular redox state as well as S-adenosyl-methionine (SAM), the main methyl donor in the body. Moreover, Hcy metabolism requires vitamins B12, folate, and B6 as cofactors. Therefore, nutritional deficiencies of any of these cofactors can elevate plasma Hcy levels resulting in hyperhomocysteinemia (HHcy)^10^. Clinical investigations provide strong evidence that HHcy is implicated in various pathological ocular disorders, including AMD, DR, macular and optic atrophy, glaucoma, corneal abnormalities and cataracts^11–13^. Various mechanisms have been reported to explain Hcy-induced visual dysfunction, such as apoptosis of retinal ganglion cells^14^, oxidative stress^15^, ischemic vascular dysregulation^16^, alteration of inflammatory mediators and extracellular matrix remodeling^17^. Our previous studies have shown that HHcy is accompanied by disruption of retinal vasculature, altered BRB with diminished tight junction proteins (ZO-I and occludin), pericyte degeneration, glia activation^18,19^, alteration of retinal pigmented epithelial cell structure and function^20^ and activation of endoplasmic reticulum (ER) stress^21^. However, the direct effect of Hcy on retinal endothelial cells and the underlying mechanisms involved in HHcy induced vascular dysfunction have not been explored. Here, we investigated the role of HHcy in BRB dysfunction to determine whether elevated Hcy directly affects retinal endothelial cells (RECs) by mechanisms that involve oxidative stress.

## Results

### Excess homocysteine disrupted retinal endothelial cell barrier function

#### Evaluation of tight junction proteins

**Figure 1:**
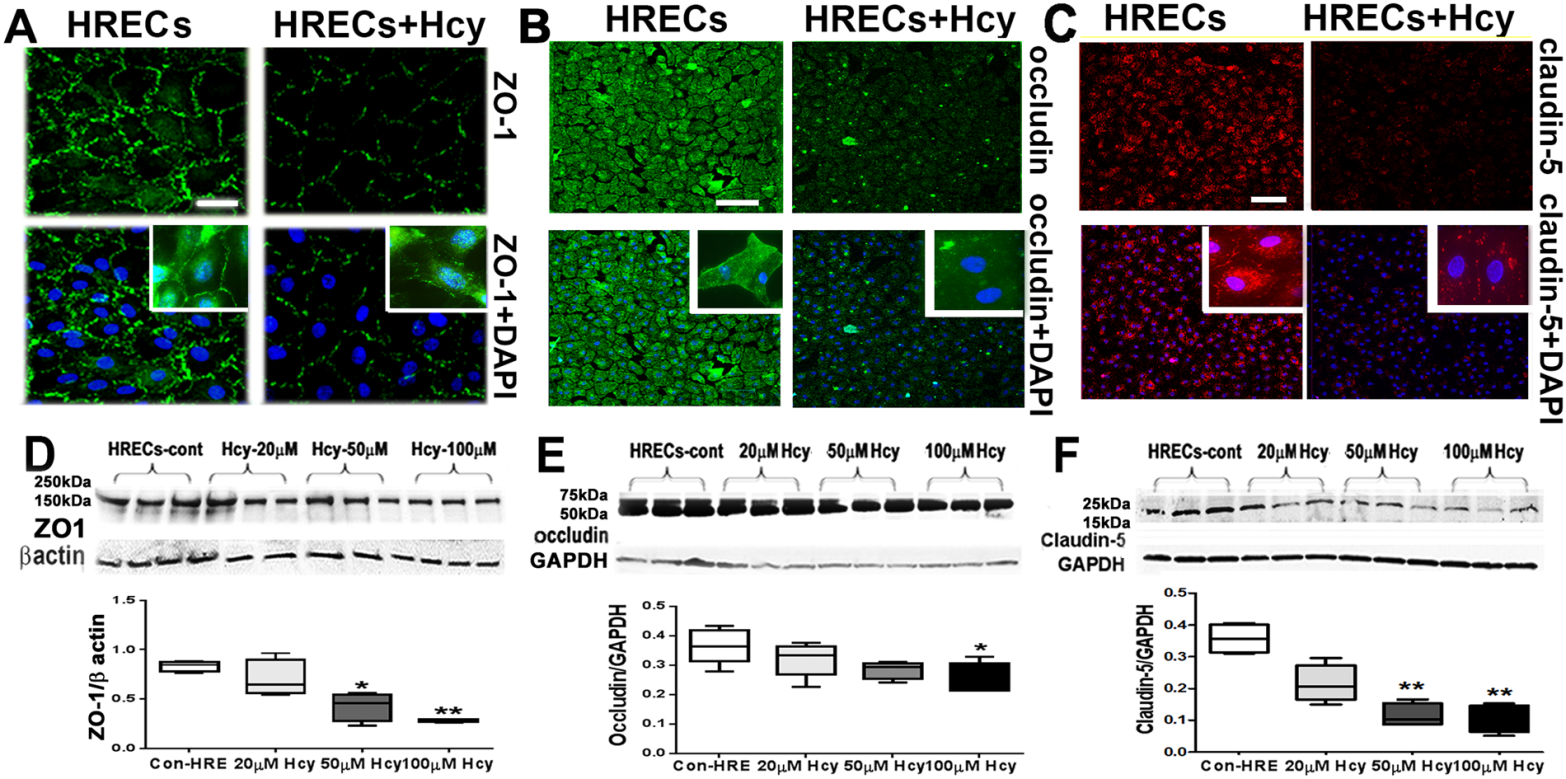
Evaluation of tight junction proteins of HRECs treated with and without Hcy-thiolactone. (**A**) Immunofluorescence of ZO-1 (green) (**B**) Immunofluorescence of occludin (green) (**C**) Immunofluorescence of claudin-5 (red) with nuclear staining DAPI (blue) in cultured HRECs subjected to treatment with Hcy (50 µM) or vehicle (PBS). Calibration bar: 50 μm. (**A**) and 100 μm. (**B** and **C**). (**D**) Western blot analysis of ZO-1 (198 KDa), (**E**) Western blot analysis of occludin (59 kD). (**F**) Western blot analysis of claudin-5 (17–26 KDa). *p < 0.05 and **p < 0.01.

The alteration we observed in BRB of the *cbs* ^−/−^ and *cbs* ^+/−^ mice^18–20^ led us to study the effect of excess Hcy on HREC barrier function (Fig. 1). The formation of intact blood–retinal barrier (BRB) by HRECs is mainly dependent on the function of tight junctions. Disassembly of tight junction proteins (TJPs) results in increased retinal vascular permeability and vascular injury^22^. HRECs were cultured in presence of 100 nM hydrocortisone to ensure high amounts of TJPs in the plasma membrane^23,24^. As shown in (Fig. 1A,B and C), continuous plasma membrane localization of ZO-1 (green), occludin(green) and claudin-5(red) staining were observed in HRECs control, while interrupted and diminished ZO-1, occludin and claudin-5 were observed between HRECs treated with 50 μM-Hcy for 18–24 hours (upper right high magnifications image). Expression of TJPs, ZO-1, occludin and claudin-5 were confirmed by Western blotting (Fig. 1D,E and F) and quantification of data from Western blotting showed significant decrease in TJP expression with Hcy treatment.

#### Transendothelial electric resistance (TER) and FITC dextran flux assay

**Figure 2:**
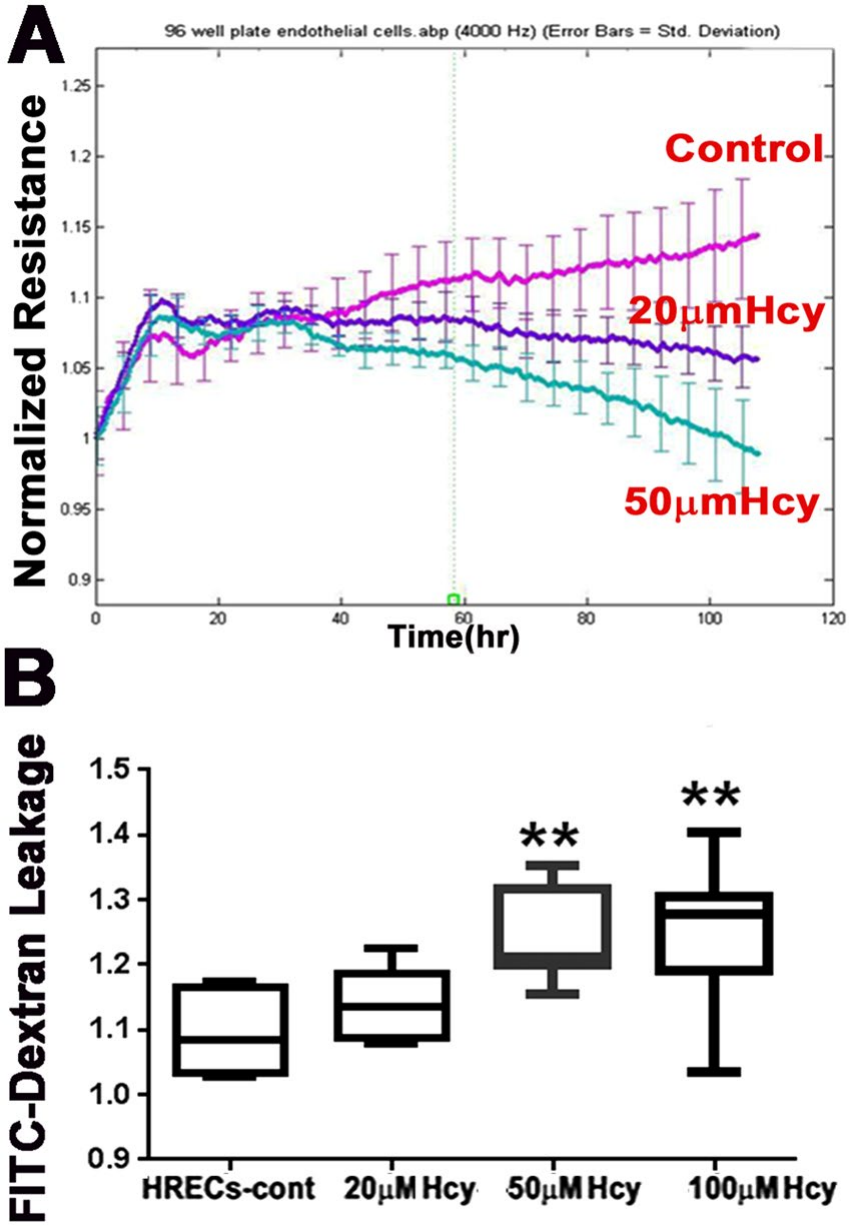
Transendothelial electric resistance (TER) and FITC dextran flux assays of HRECs cells treated with and without Hcy-thiolactone. (**A**) TER and (**B**) FITC dextran flux through the confluent monolayer in the Hcy-treated endothelial cells. **p < 0.01.

Functional assays were performed to investigate whether HHcy disrupted barrier function in HREC monolayers using real-time analysis of transendothelial electric resistance (TER). Cells treated with different concentrations of Hcy (20 and 50 µM) showed significant reductions in TER compared to vehicle treated HREC monolayers (Fig. 2A). To confirm the role of excess Hcy in altering the barrier function of HREC monolayers, we explored whether HHcy treatment induces permeability changes to FITC dextran flux through the confluent monolayer. HREC monolayers treated with HHcy showed significantly increased permeability to FITC-dextran in a dose-dependent manner as indicated by increasing diffusive flux (Po) for FITC-dextran (Fig. 2B).

### Examination of Hcy on HRECs angiogenesis and cell viability

#### Effect of Hcy on angiogenic potential of HRECs

**Figure 3:**
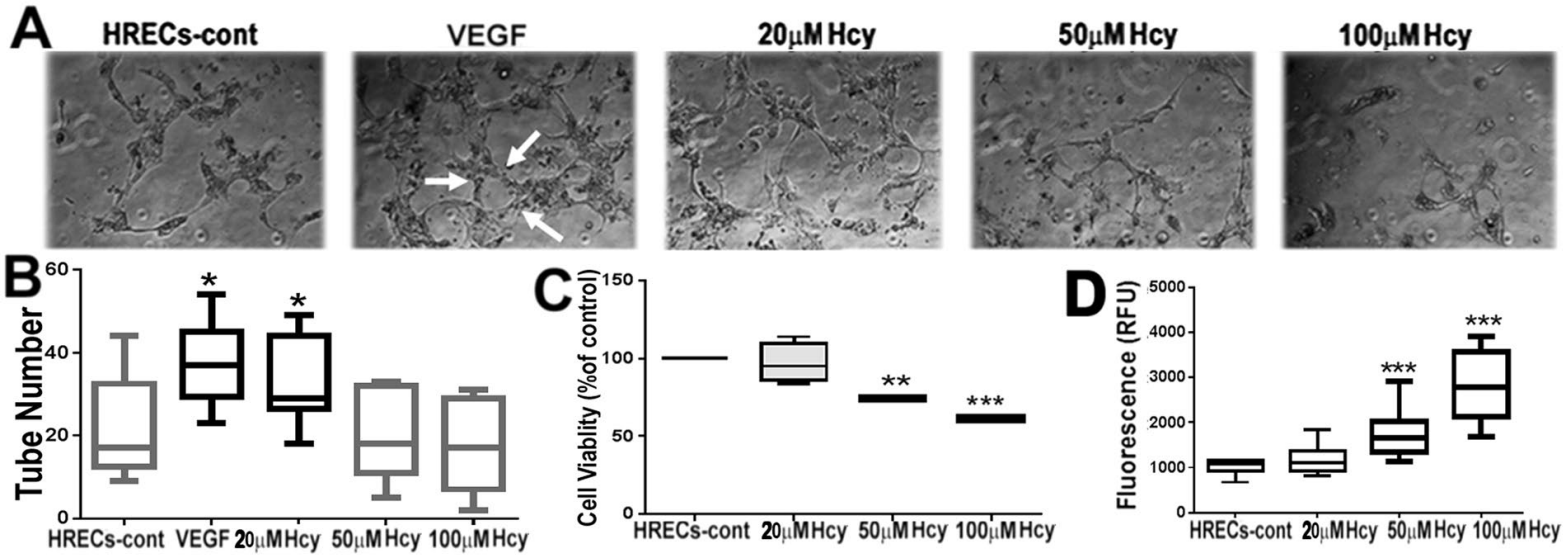
Evaluation of antigenic potential and cell viability of Hcy-treated HRECs. Cell were treated with different concentrations of Hcy and VEGF was used as a positive control. (**A**) Tube formation assay of HRECs, white arrows indicate tube formation. (**B**) Assessment of tube number. (**C**) Cell Viability (MTT) assay. (**D**) Apoptosis (Caspase3/7) assay. Error bars indicate SD from the mean, n = 3.

Endothelial cells are the principal cells involved in retinal neovascularization. Endothelial cells proliferate to provide the necessary number of cells for making new vessels. Subsequent to this proliferation, the new outgrowth of endothelial cells must reorganize into a three-dimensionally tubular structure. We evaluated the effects of different concentrations of Hcy on tube formation by HRECs. Figure 3A shows HRECs with no treatment, HRECs treated with VEGF (50 ng/ml) as a positive control, and HRECs treated with 20, 50, or 100 µM Hcy (18–24 h). 20 μM of Hcy treatment significantly increased the number of tubes formed in HRECs (as indicated by white arrows) in a manner similar to that observed with VEGF treatment. Interestingly, higher concentrations of Hcy (50 or 100 µM) did not increase the number of tubes formed.

#### Effect of Hcy on HRECs Viability

To further investigate the effect of excess Hcy on cell viability and cell death (apoptosis), HRECs treated with and without different concentrations of Hcy (20, 50 and 100 µM) were subjected to MTT assay (Fig. 3C) and detection of Caspase 3/7 (Fig. 3D). HRECs showed a significant decrease in cell viability and significant increase in cell death in HRECs treated with 50 µM and 100 µM of Hcy. Taken together, the data suggests that higher concentrations of Hcy (50 µM and 100 µM) decrease HREC viability and increase cell death.

### Hyperhomocysteinemia Induced Activation of Oxidative Stress

#### Increased retinal ROS production in a mouse model of hyperhomocysteinemia

**Figure 4:**
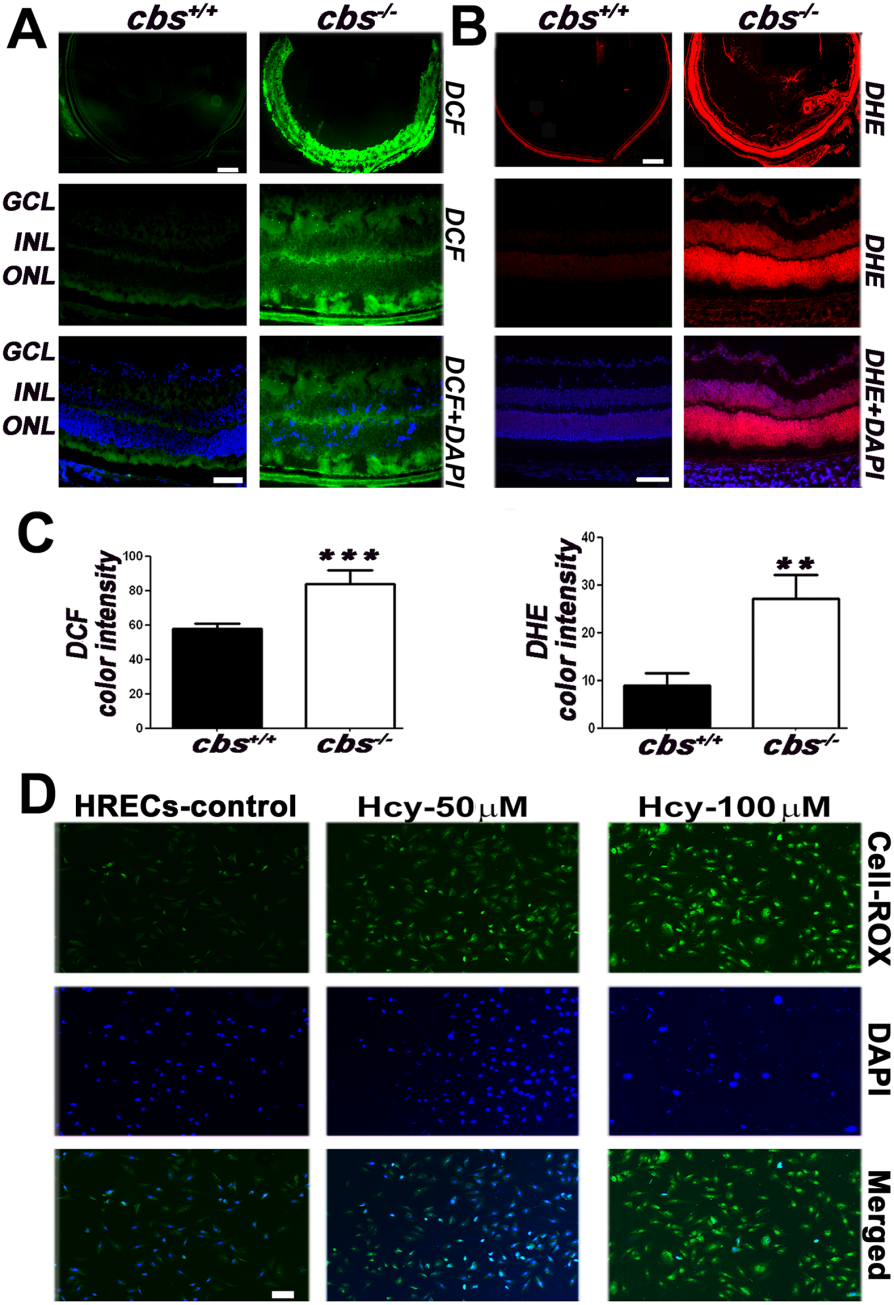
Evaluation of ROS production induced by Hyperhomocysteinemia. (**A**) DCF staining (green) of the *cbs* ^+/+^and *cbs* ^−/−^ retinas. (**B**) DHE staining for superoxide (red) in the *cbs* ^+/+^ and *cbs* ^−/−^ retinas. (**C**) Quantification of the color intensity of DCF and DHE staining, **p < 0.01 and ***p < 0.001. (**D**) Cell ROX green staining for ROS in HRECs treated with and without Hcy (green).

ROS production was evaluated *in vivo* using *cbs* ^+/+^ and *cbs* ^−/−^mice (Fig. 4A,B and C) and *in vitro* using HRECs treated with and without different concentrations of Hcy (50 and 100 μM) as shown in (Fig. 4D). Retinal cryosections from *cbs* ^+/+^ and *cbs* ^−/−^ mice stained with DCF (Fig. 4A) and DHE (Fig. 4B) showed marked increases in ROS and superoxide production in the *cbs* ^−/−^ mice compared to the *cbs* ^+/+^ controls. Quantification of color intensity revealed a significant increase in ROS and superoxide production in the *cbs* ^−/−^mice (Fig. 4C). HRECs treated with Hcy had increased ROS production compared to non-treated HRECs (Fig. 4D).

#### Effect of hyperhomocysteinemia on retinal anti-oxidant capacity

**Figure 5:**
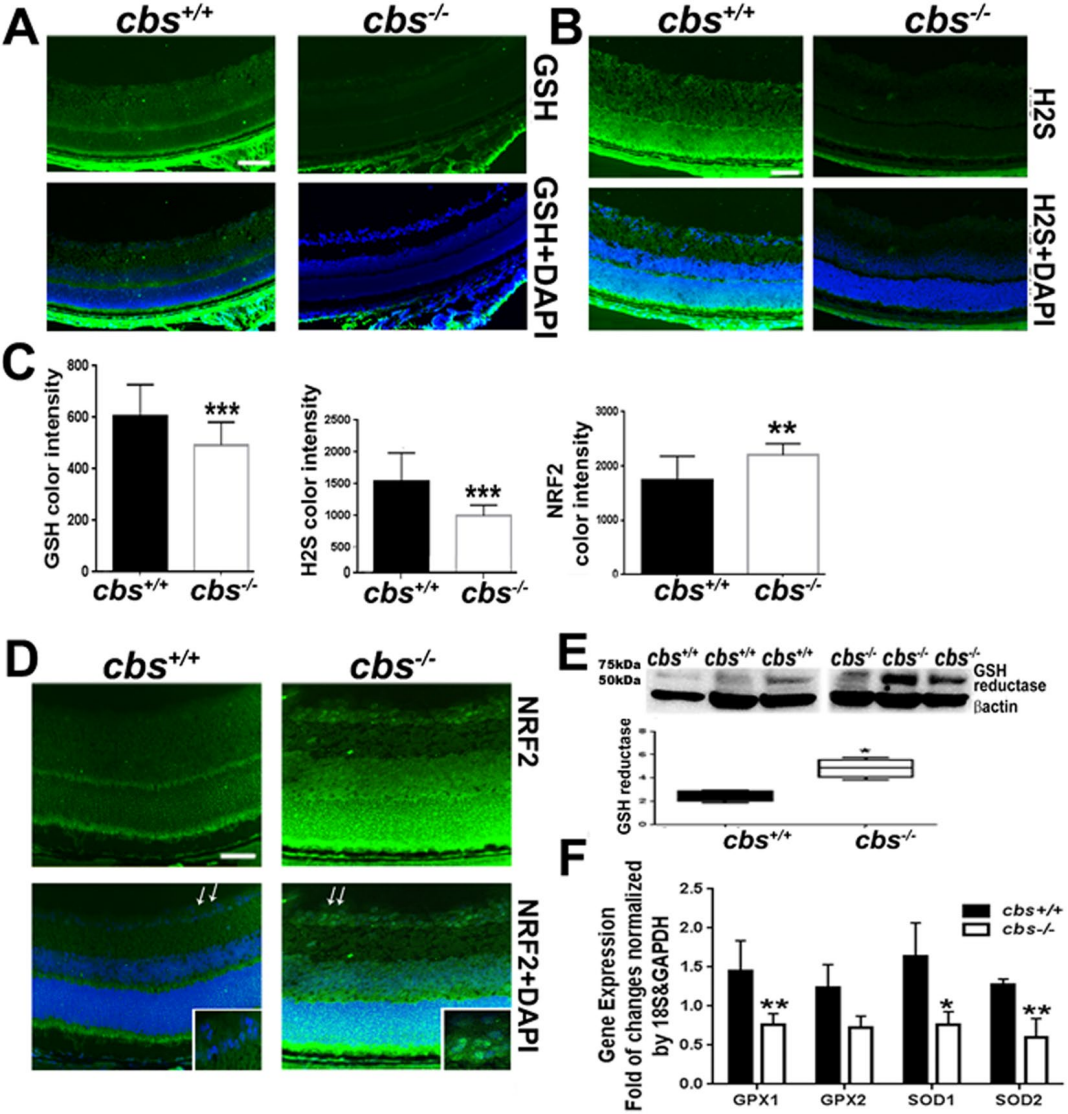
Evaluation of the retinal antioxidant capacity of the hyperhomocysteinemic (*cbs* ^−/−^) mice. (**A**) Retinal frozen sections from the *cbs* ^+/+^ and *cbs* ^−/−^ mice immunostained for GSH (green). (**B**) Retinal frozen sections from the *cbs* ^*+/+*^ and *cbs* ^−/−^ mice stained for H_2_S (green). (**C**) Quantification for the color intensity of GSH, H_2_S and NRF2. (**D**) Retinal frozen sections from the *cbs* ^*+/+*^ and *cbs* ^−/−^ mice immunostained for NRF2 (green). (**E**) Western blot analysis of GSH reductase expression in the *cbs* ^−/−^ retina compared to control retina. (**F**) RT-PCR evaluation of anti-oxidant gene expression in the *cbs* ^−/−^ retina compared to control retina. Calibration bar is 50 μm.

The anti-oxidant capacity in retina was evaluated in the *cbs* ^−/−^mice (Fig. 5) by measuring the level of glutathione (Fig. 5A), H2S (Fig. 5B) and NRF2 (Fig. 5D) by IF staining of retinal frozen sections. Our results showed a significant decrease in the levels of GSH and H_2_S and an increase in the NRF2 levels in cbs^−/−^ mice compared to cbs^+/+^ control mice. Color intensity was assessed using ImageJ and quantified using Graphpad Prism software as shown in Fig. 5C. Furthermore, Western blot analysis confirmed that glutathione reductase levels were significantly increased in cbs^−/−^ retinas but not in cbs^+/+^ control retinas (Fig. 5E), indicating less GSH, presumably, to compensate for increased ROS production. In addition, mRNA expression of antioxidant genes superoxide dismutase (SOD1 and SOD2) and glutathione peroxidase (GPX1 and GPX2) were decreased in the cbs^−/−^ retinas compared to in cbs^+/+^ control retinas (Fig. 5F).

**Figure 6:**
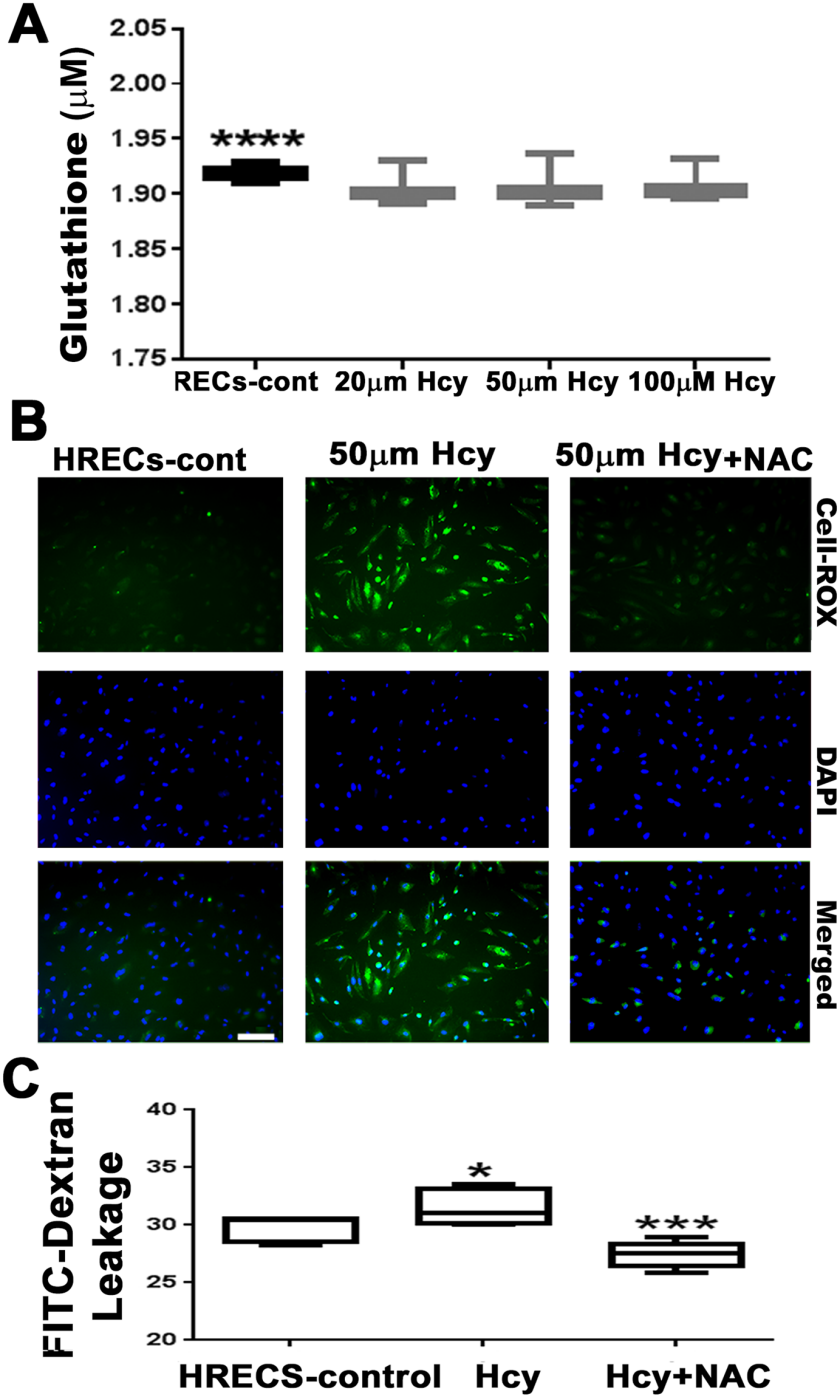
Evaluation of the antioxidant capacity of HRECs treated with and without Hcy-thiolactone. (**A**) Measurement of GSH activity in HRECs treated with Hcy (20, 50 and 100 μM) *p < 0.05. (**B**) IF staining of ROS (green) in HRECs treated with 50 μM of Hcy with and without the addition of anti-oxidant NAC, (50 mM). Calibration bar; 100 μm. (**C**) FITC-dextran assay illustrating fluorescein leakage in HRECs treated with 50 μM of Hcy, with and without the addition of 50 mM of NAC, *p < 0.05.

The effect of excess Hcy on the redox state of HRECs was also evaluated *in vitro*. HRECs treated with and without different concentrations of Hcy (20, 50 and 100 μM) were evaluated for GSH activity, which showed significant reduction in GSH activity in Hcy treated cells compared to non-treated cells (Fig. 6A). To confirm our findings further, HRECs were treated with 50μM-Hcy with and without the anti-oxidant NAC (50 mM) and were evaluated for ROS production (Fig. 6B). NAC was able to block the ROS production induced by Hcy treatment in HRECs. Furthermore, we evaluated whether NAC exposure would alter Hcy induced HREC permeability by measuring the level of FITC-dextran leakage. We found a significant increase in FITC-dextran leakage in Hcy-treated cells, which was attenuated by NAC treatment (Fig. 6C).

## Discussion

In our previous studies, we showed that elevated Hcy resulted in altered retinal vasculature as evidenced by ischemia, neovascularization, vascular leakage and a deficient blood-retinal barrier^18–20^. In this study, we extended our investigation by studying the direct effect of Hcy on HRECs, the functional unit of vascular BRB, with an emphasis on possible underlying molecular mechanisms. Important components of the endothelial BRB are tight junction proteins (TJPs). TJPs are specialized highly selective multiprotein complexes which function as a tight seal for intercellular spaces, generating a permeability barrier that regulates the flux of fluids and solutes^25^. Among TJPs, the ZO family and occludin have been reported as crucial components of tight junctions in retinal endothelial cells^26^. Compromised tight junctions in endothelial cells are associated with breakdown of BRB, a prominent pathophysiological event in various retinal diseases. Altered expression of ZO-1 and occludin are well-established contributors to breakdown of BRB^4^. Herein, we found that Hcy reduced ZO-1, occludin and claudin-5 protein levels in HRECs, suggesting that Hcy adversely affects the tightness of the retinal endothelial barrier.

Normal cellular expression and organization of tight junction proteins is crucial for preservation of retinal endothelial barrier function. Indeed, the number and complexity of tight junction proteins is inversely related to barrier permeability^27^. Therefore, it is not surprising that our results demonstrated that Hcy treatment reduced permeability of HRECs as evidenced by decreased TER as well as increased FITC dextran leakage. Of note, Hcy was previously reported to increase permeability of brain endothelial cells via modulating tight junctions^28^.

The relationship between Hcy and angiogenesis remains controversial. We previously demonstrated that elevated Hcy levels resulted in prominent features of angiogenesis *in vivo* ^18^. However, a handful of studies reported angiostatic effects of Hcy in vascular biology^8,29^. In the present study, a lower concentration of Hcy increased tube formation, however, higher concentrations decreased tube formation by HRECs. These results could be explained by the cytotoxic effect of higher concentrations of Hcy on HRECs as evidenced by the dose dependent decrease in cell viability. These results are in agreement with previous studies that reported Hcy-induced growth inhibition, dysfunction and apoptosis in human microvascular endothelial cells and cerebral endothelial cells^30–32^. Various mechanisms have been suggested for Hcy-induced apoptosis of endothelial cells including the mitochondrial apoptotic pathway, endoplasmic reticulum stress pathway and death receptor pathway^21^. Our results showed that Hcy impacted the mitochondrial apoptotic pathway, inducing a dose-dependent increase in caspase 3/7 activity in HRECs. Our previous studies of the cbs^−/−^ mouse retina reported significant increase in the levels of cleaved caspase-3 and cleaved PARP. Interestingly, some of the cleaved caspase-3 cells in stained retinal cryosections showed co-localization with isolectin-B4, a marker for blood vessels, suggesting apoptosis in the REC of the hyperhomocysteinemic mice^19^. Our results are consistent with the notion that excess Hcy treatment increases cleaved caspase-3 expression in human endothelial cells and this effect is inhibited by modulating oxidative stress^33^.

To gain insight into the underlying mechanisms of our findings, we examined the effect of HHcy on oxidative stress both *in vivo* and *in vitro*. A large body of evidence suggests a strong association between Hcy and oxidative stress in various diseases^34^ In ocular diseases, HHcy has been found to induce death of lens epithelial cells in cataract and loss of RGCs in glaucoma through mechanisms involving ROS production^14,35^. Moreover, oxidative stress has been suggested as major player in the adverse effects of Hcy on endothelial function^36^. Hcy increases ROS generation in both endothelial and smooth muscle cells due to metabolic processes or due to endothelial injury^37^. In this context, several studies report that Hcy promotes the production of hydrogen peroxide, superoxide anion and hydroxyl radicals via self-oxidation of its highly reactive sulfhydryl group and/or down regulation of cellular antioxidant defenses. These events lead ultimately to oxidative inactivation of NO production and function and thus endothelial dysfunction^38,39^. In the present study, we found that elevated Hcy levels induced oxidative stress in retinas of *cbs* ^*−/−*^ mice. Moreover, retinas of CBS deficient mice showed decreased levels of the antioxidant markers, SOD1, SOD2, GPX1, GPX2 and the main antioxidant in the retina GSH. However, there were increases in the levels of glutathione reductase and NRF2. NRF2 regulates the expression of antioxidant proteins that protect against oxidative damage. The increased levels of NRF2 and glutathione reductase were presumably insufficient to compensate for the increased ROS production. *In vitro*, Hcy-treated HRECs showed a significant decrease in GSH and a dose-dependent increase in ROS levels. Interestingly, the Hcy-induced increase of FITC dextran leakage in HRECs was abolished by co-treatment with the antioxidant NAC, suggesting oxidative stress as a potential player in Hcy-induced retinal endothelial hyperpermeability and dysfunction.

Another potential mechanism for Hcy-induced oxidative endothelial damage is reduced hydrogen sulfide (H_2_S) levels. Elevated Hcy may reduce availability of systemic cysteine and decreaset he enzymes responsible for conversion of cysteine to H_2_S especially cystathionine γ-lyase^40^. H_2_S is a gasotransmitter recently recognized as a cytoprotective mediator that regulates vascular homeostasis and preserves the endothelium from oxidative stress and inflammation. H_2_S is endogenously synthesized by three enzymes: cystathionine γ-lyase, 3-mercaptopyruvate sulfurtransferase and CBS^41^. Both cystathionine γ- lyase and CBS require vitamin B6 as a cofactor. Therefore, HHcy that results from vitamin B6 deficiency is associated with impaired H_2_S synthesis^42^. As we used deficiency of CBS as a model for our HHcy, it was expected that retinas from *cbs* ^*−/−*^ would show reduced H_2_S levels. Of note, disturbed H_2_S bioavailability has recently been recognized as a hallmark of endothelial dysfunction. H_2_S protects the endothelium via various mechanisms including modulation of oxidative stress and inhibition of inflammation and leukocyte adhesion^40,41^.

In summary, although animal studies have shown that antioxidants have beneficial effects on the development of retinopathy and antioxidants are being used for other chronic diseases however, there are very limited clinical trials using antioxidants in treatment of retinopathy^43–45^.

The results of this study support our previous findings that Hcy exerts detrimental effects on BRB and provides compelling evidence of direct damaging effects of Hcy on function and structure of retinal endothelial cells. Our results show that elevated Hcy increase ROS formation and decrease the anti-oxidant capacity *in vivo* and *in vitro*, which leads us to suggest increased oxidative stress as a potential mechanism of Hcy-induced retinal hyperpermeability and endothelial dysfunction. The findings support investigation of antioxidants as therapeutic strategies for retinopathies associated with excess Hcy such as DR and AMD.

## Materials and Methods

### Animal details

Wild-type (*cbs* ^+/+^) and homozygous mutant (*cbs* ^−/−^) mice (B6.129P2-Cbstm1Unc/J; Jackson Laboratory, Bar Harbor, ME) were used at ages 3–5 weeks as the *cbs* ^−/−^ mice have a shortened life span ~3–5 weeks. Mice genotyping, housing conditions and husbandry were as previously described^46^. For the present experiments, all experimental procedures complied with Augusta University animal care guidelines, the Public Health Service Guide for the Care and Use of Laboratory Animals and the Association for Research in Vision and Ophthalmology Statement for Use of Animals in Ophthalmic and Vision Research. All studies were approved by the appropriate institutional animal review board (Augusta University Animal Care and Use Committee: IACUC Approval for Protocol 2014–0683).

### Cell culture

Normal plasma Hcy levels are between 10–12 µmol/L. HHcy has been classified into moderate (15 to 30 µmol/L), intermediate (30 to 100 µmol/L), and severe (greater than 100 µmol/L). Human retinal endothelial cells (HREC) and complete medium (including supplements, growth and attaching factor) were obtained from Cell Systems (Kirkland, WA) and used as an endothelial model system to test the effect of different levels of Hcy on human retinal endothelial cells (HRECs). Purity of HRECs was confirmed by Immunofluorescence staining for endothelial cell marker CD31 and pericytes marker alpha-smooth muscle actin. HRECs were positive for CD31but negative for alpha-smooth muscle actin (Supplementary Figure). Cells were grown in Endothelial Basal Medium −2 (EBM-2) (LONZA New Orleans, LA, USA) containing hydrocortisone, and used between 5–7 passages. At 80–90% confluence, HREC were transferred to serum starved-medium (1% serum) for 24 hours incubation prior to treatment with or without Hcy thiolactone (20, 50, or 100 μM) followed by studies to evaluate REC barrier function, angiogenic capacity, and reactive oxygen species (ROS) formation as described below.

### Immunofluorescent assessment of tight junction proteins and oxidative stress markers

HRECs were plated at 1 × 10^5^ in 8-well chamber slides (Sigma-Aldrich Chemical Corp., St. Louis, MO, USA) and treated with and without Hcy (20, 50, or 100 μM) for 24 hr. Cells were then fixed with 4% formalin for 10 min, washed with PBS, and blocked with Power Block (BioGenex, Fremont, CA, Ca. #BS-1310–25) for 1 hr. Thereafter, the cells were incubated at 4 °C overnight with Antibodies for ZO-1(Abcam, Cambridge, Massachusetts, USA, Cat.# ab59720), occludin (Invitrogen, Eugene, Oregon, USA, Mouse monoclonal Cat.# 33–1500), claudin-5 (Invitrogen, Eugene, Oregon, USA, Rabbit Polyclonal Cat.# 34–1600), anti-GSH-1 (Santa Cruz, Dallas, Texas, USA. Cat.# sc-292189), anti-Nrf2 (Abcam, ab137550), anti CD31 (Novus Biologicals, NB100–2284) and anti α-smooth muscle actin (Abcam, ab5694). After primary antibody treatment, cells were then washed 3 times with PBS containing 0.3% Triton-X and incubated with appropriate secondary antibodies (Alexafluor and Texas red avidin, Invitrogen). Slides were cover-slipped using Fluoroshield containing DAPI (Sigma-Aldrich) as a counter stain. Images were captured by fluorescent microscopy (Carl Zeiss, Göttingen, Germany).

### Protein extraction and Western blot analysis

Separated neuronal retinal tissues and cultured HRECs were homogenized in modified RIPA buffer supplemented with 1:100 (v/v) of proteinase/phosphatase inhibitor cocktail (Thermo Scientific). Equal amounts of protein were denatured by boiling in Laemmli sample buffer, resolved by sodium dodecyl sulfate-polyacrylamide gel electrophoresis using gradient gel (4 to 20%, Pierce, Rockford, IL), transferred to nitrocellulose membrane, and then reacted with ZO-1(Abcam, ab59720), occludin (Abcam,ab31721), Claudin-5 (Invitrogen, Eugene, Oregon, USA, Rabbit Polyclonal Cat.# 34–1600), albumin (Bethyl, TX, USA), GAPDH (Sigma-Aldrich), or glutathione reductase antibody (Abcam, ab16801) at 4 °C overnight. This was followed by incubation of blots with horseradish peroxidase–conjugated antibody and then enhanced chemiluminescence (Thermo Scientific) was used for protein visualization. Intensity of immunoreactivity was quantified by densitometry using ImageJ software (NIH).

### Electric Cell-Substrate Impedance Sensing (ECSIS)

HRECs were grown in 96-well electrode arrays (96W20idf PET, Applied Biophysics Inc.) and the electric currents passing through the monolayers were measured independently in each chamber. HRECs were seeded at a density of 5 × 10^4^ cells/chamber. Once confluent (Capacitance <20nF), the cells were serum-starved and treated with Hcy (20 μM and 50 μM) for 24 hours. Transendothelial resistance (TER) was recorded over the experimental time course. Integrity of the endothelial monolayer was confirmed microscopically at the end of each experiment and also by a final TER measurement. Resistance values for each chamber were normalized as the ratio of measured resistance to baseline resistance (normalized resistance).

### Fluorescein isothiocyanate (FITC)-dextran permeability assay

HRECs were grown to confluence on collagen/fibronectin-coated membranes with 0.4μm pores (Transwell; Corning Costar). FITC-dextran flux permeability assays were then performed following our previously described method^20^. Briefly, cells were serum starved for 24 hours before Hcy treatment (20, 50, or 100 μM) (Sigma-Aldrich) for 16–24 hours to the upper chambers in the presence or absence of an oxidative stress inhibitor NAC (5 mM), followed by addition of 10 μM FITC-dextran to the upper chambers. Aliquots were collected from the upper and lower chambers at 1, 3, or 6 hours then placed in a 96-well plate to measure the fluorescence intensity with a plate reader. The rate of diffusive flux (Po) FITC-dextran was calculated by the following formula: Po = [(FA/∆t)VA]/(FLA)(24). Where Po is in centimeters per second; FA is lower chamber fluorescence; FL is upper chamber fluorescence; ∆t is change in time; A is the surface area of the filter (in square centimeters); and VA is the volume of the lower chamber (in cubic centimeters).

### Quantitative Reverse-Transcriptase Polymerase Chain Reaction (RT-q PCR)

Retinas were isolated from *cbs* ^+/+^ and *cbs* ^−/−^ mice. Total RNA was extracted using TRIzolTM Reagent (Invitrogen, Eugene, Oregon, USA). After quantification, 2 μg of RNA was reverse transcribed using iScript™ Synthesis kit (BioRad Laboratories, Hercules, CA). The cDNA was amplified using Absolute QPCR SYBR Green Fluorescein Mix (Thermo Scientific, Surrey, UK), the BioRadiCycler (BioRad, Hercules, CA) and gene specific primers. The primers used were GAPDH (CAT GGC CTC CAA GGA GTA AGA), 18S (AGT GCG GGT CAT AAG CTT GC), GPX1 (GAG GAA CAA CTA CCC GGG ACT A) and SOD1 (TGG GTT CCA CGT CCA TCA GTA). Amplification parameters were as follows: 40 cycles of 95 °C for 30 seconds, 60 °C for 30 seconds, and 72 °C for 30 seconds. The purity of the end products was confirmed using melt curve analysis. 18 S and GAPDH were used for normalization and comparative CT method was used to obtain fold changes in gene expression^18^.

### Measurement of superoxide by dihydroethidium (DHE)

The oxidative fluorescent dye DHE was used to measure superoxide anion in fresh frozen eye sections per our previously described method^47^. Briefly, cryosections were incubated with 2 μM DHE (Sigma-Aldrich) for 30 minutes at 37 °C and then observed using a fluorescence microscope (Carl Zeiss Optical, Germany, excitation 518 nm, emission 605 nm). DHE is oxidized by superoxide anion to ethidium that binds with DNA in the nucleus producing red fluorescence. The fluorescence intensity was measured using ImageJ software (NIH).

### Measurement of ROS activity by DCF and CellROX^®^ Green Reagent

2, 7-dichlorodihydro-fluorescein diacetate (DCFH-DA) is oxidized by cellular ROS to Dichlorofluorescein (DCF) that emits a green fluorescent signal. OxiSelect ROS Assay Kit (Cell Biolabs, San Diego, CA) and CellROX® Green Reagent (Invitrogen, Grand Island, NY, USA) were used for detection of oxidative stress by measuring ROS activity. In brief, retinal sections or HRECs treated with Hcy, were incubated with DCFH-DA or 5 μM CellROX® Green Reagent for 30 min at 37 °C to detect intracellular ROS. Additional experiments were performed in which HRECs were treated with Hcy (50 μM) in the presence or absence of NAC (50 μM). ROS formation was measured by fluorescence microscopy, and the fluorescence intensity was measured using ImageJ software.

### *In vitro* endothelial tube formation on Matrigel

An assay to evaluate tube formation by endothelial cells was performed as previously described^48^. Briefly, HRECs were incubated with Hcy (20, 50 and 100 μM) at 37 °C overnight in Matrigel-coated culture plates. VEGF 50 ng/ml (BD Bioscience) was used as positive control. Tube formation was observed using an inverted microscope. The images were captured with a digital camera attached to the microscope (ZEISS Axioverst 40 CFL). The degree of tube formation was further quantified by measuring the total length of tube-like cells using ImageJ software.

### Assessment of Cell viability and Cell death by MTT and Caspase 3/7 assay

Viability of HRECs after treatment with Hcy (100, 50 and 20 µM) was determined using MTT Cell Proliferation Assay Kit (Invitrogen, Grand Island, NY, USA). HRECs were grown on 96-well plates at a density of 2 × 10^4^ cells/well in phenol red free medium and incubated with different concentrations of Hcy for 24 hours. The medium was removed and the cells were treated with 20 μL of MTT (5 mg/mL) and incubated for 4 h hours at 37 °C prior to the addition of 100 μL of DMSO. The resulting formazan product was measured spectrophotometrically at 540 nm using a microplate reader (VERSA max, Molecular Devices, Sunnyvale, CA, USA).

Caspase-3/7 activity in homocysteine treated HRE cells was measured using the Apo-ONE Homogenous caspase-3/7 system according to the manufacturer’s instructions (Promega). The intensity of the emitted fluorescence was determined at an excitation wavelength range of 485 ± 20 nm and an emission wavelength range of 530 ± 25 nm using microplate reader (VERSA max, Molecular Devices, Sunnyvale, CA, USA). Caspase-3/7 activity was expressed as net fluorescence intensity (RFU). Data shown are means ± SD of three different independent experiments.

### Assessment of hydrogen sulfide (H_2_S)

H_2_S was detected in fresh frozen eye sections (10 μm) using Hydrosulfide Naphthalimide-2 (HSN2), a selective fluorescent probe for H_2_S^49^. In brief, retinal sections were fixed with 4% paraformaldehyde, then washed three times with PBS and incubated with the probe for 1 hr. The slides were subsequently washed with PBS, mounted with DAPI and immunofluorescence visualized using a fluorescent microscope (Carl Zeiss, Göttingen, Germany).

### Assessment of glutathione (GSH)

GSH levels in the HRECs extracts were measured using GSH-Glo™ Glutathione Assay kit (Promega, Madison, WI, USA) according to the manufacturer’s instructions. GSH-Glo™ reagent was added to each well and mixed on a plate shaker at room temperature for 30 minutes. After adding Luciferin detection reagent for 15 minutes, luminescence was measured using plate reader. GSH concentration was calculated using a standard curve obtained from GSH standard solutions.

### Data analysis

The results are conveyed as mean ± SD. Differences amongst experimental groups were assessed by using the two-tailed *t* test or one way analysis of variance (ANOVA). When statistical differences were detected using ANOVA, a post hoc Tukey’s test was performed to determine which groups differed. A confidence level of P < 0.05 was considered statistically significant.

## Electronic supplementary material

Supplementary figure 1
