# Supplementary material for: Hyperhomocysteinemia Alters Retinal Endothelial Cells Barrier Function and Angiogenic Potential via Activation of Oxidative Stress
Source: Sci Rep. 2017 Sep 20;7:11952. doi: 10.1038/s41598-017-09731-y (PMC5607263; doi:10.1038/s41598-017-09731-y)
Supplement: Supplementary file 1 — Supplementary figure 1 [file 41598_2017_9731_MOESM1_ESM.rtf]

Hyperhomocysteinemia Alters Retinal Endothelial Cells Barrier Function and Angiogenic Potential via

Activation of Oxidative Stress


Riyaz Mohamed 1,2, Isha Sharma1,2 , Ahmed S Ibrahim1,2,3 , Heba Saleh1,2, Nehal M Elsherbiny1,2,3, Sadanand Fulzele6 , Khaled Elmasry1,2,4 Sylvia B. Smith2,4,5, Mohamed Al-Shabrawey1,2,4,5, Amany Tawfik1,2,4,5*

1Department of Oral Biology and Anatomy, Dental College of Medicine, Augusta University, Augusta,  GA, USA;  2James and Jean Culver Vision Discovery Institute, Medical College of Georgia (MCG), Augusta University, USA; 3Department of Biochemistry, Faculty of Pharmacy, Mansoura University, Mansoura, Egypt; 4Department of Cellular Biology and Anatomy, MCG, Augusta University, Augusta, GA, USA; 5Department of Ophthalmology, MCG, Augusta University, Augusta, GA, USA; 6Department: Orthopedic Surgery, MCG, Augusta University, Augusta, GA, USA
Short running head: Hyperhomocysteinemia alters Retinal Endothelial Cells.


*Corresponding author:


Amany Tawfik, M.D.

Department of Oral Biology and Anatomy

Dental and Medical Colleges of Georgia,

James and Jean Culver Vision Discovery Institute,

Augusta University, 1120 15th Street, CB 1114, Augusta, GA 30912-2000

706-721-2582 (Phone)

706-721-9415 (Fax)

Email: amtawfik@augusta.edu

Supplementary Figure 1:


Supplementary Figure 1: Immunofluorescence staining of HRECs for endothelial cell marker CD-31 (red) and pericyte marker alpha-smooth muscle actin (green). Image showing that HRECs are positive for CD31 but negative for á-smooth muscle actin indicating HRECs free from retinal pericyte
